# Protecting the vulnerable: addressing the COVID-19 care needs of people with compromised immunity

**DOI:** 10.3389/fimmu.2024.1397040

**Published:** 2024-05-02

**Authors:** Raymund R. Razonable

**Affiliations:** ^1^ Division of Public Health, Infectious Diseases and Occupational Medicine, Department of Medicine, Mayo Clinic, Rochester, MN, United States; ^2^ William J. von Liebig Center for Transplantation and Clinical Regeneration, Mayo Clinic, Rochester, MN, United States

**Keywords:** COVID-19, immunocompromised, SARS-CoV-2, prevention, treatment

## Abstract

While the general population regained a certain level of normalcy with the end of the global health emergency, the risk of contracting COVID-19 with a severe outcome is still a major concern for people with compromised immunity. This paper reviews the impact of COVID-19 on people with immunocompromised status, identifies the gaps in the current management landscape, and proposes actions to address this unmet need. Observational studies have demonstrated that people with immune dysfunction have a higher risk of COVID-19–related hospitalization and death, despite vaccination, than the general population. More research is needed to define the optimal prevention and treatment strategies that are specific to people with immunocompromised status, including novel vaccination strategies, monoclonal antibodies that provide passive immunity and complement suboptimal vaccination responses, and improved and safer antiviral treatment for COVID-19. Preventive measures beyond vaccination alone are urgently needed to protect this vulnerable population.

## Introduction

Rapid medical advances and increased scientific understanding have led to improvements in the management of COVID-19; however, people with compromised immunity remain at increased risk of contracting SARS-CoV-2 and experiencing severe outcomes, including hospitalization and death (1). This heterogenous population comprises approximately 6.6% of US adults (2), and includes people with solid and hematologic cancers, advanced HIV, primary immunodeficiencies, and those taking immunosuppressive drugs for transplantation or autoimmune diseases (3–6). Despite vaccination, people with compromised immunity have been disproportionately affected by the COVID-19 pandemic, with negative consequences in terms of individual and societal costs (1, 7, 8). In addition to immunocompromised status, these individuals often have advanced age and other comorbidities that increase their risk for poor outcomes (7–11).

Data are lacking to guide optimal prevention and treatment strategies for COVID-19 in immunocompromised individuals. Furthermore, there is a need for individualized therapy because of the broad range of immune response abnormalities and their underlying illnesses. This paper reviews the impact of COVID-19 on people with immunocompromised status, identifies the gaps in the current management landscape, and proposes actions to address this unmet need.

## Impact of COVID-19 on people with immunocompromised status

Since its start, the COVID-19 pandemic has caused more than 7 million deaths worldwide (12). Despite the availability of effective vaccines and treatments, SARS-CoV-2 continues to take a toll. In the United States alone, the Centers for Disease Control and Prevention (CDC) estimated that COVID-19 led to approximately 911,000 new hospital admissions and 74,000 attributable deaths in 2023 (13).

### Clinical impact: increased disease severity and mortality

Three large observational studies attempted to quantify the increased risk and severity of COVID-19 in immunocompromised populations, COVID-19–Associated Hospitalization Surveillance Network (COVID-NET), Emerging Populations and Outcomes associated with COVID-19-Health Conditions (EPOCH-US), and INFORM (1, 7, 8).

COVID-NET monitored cases from March 1, 2020, through February 28, 2022, and included a representative sample of 22,345 adults aged ≥18 years in the United States hospitalized with COVID-19. Data from this population-based active surveillance suggested that immunocompromised people were overrepresented, accounting for 12% of adult hospitalizations (8). Furthermore, among vaccinated adults, those with immunocompromised status had higher risks of intensive care unit (ICU) admission (adjusted odds ratio 1.40) and in-hospital death (1.87) compared with those with competent immune function.

The EPOCH-US study included a cohort of over 16 million people registered in the Healthcare Integrated Research Database from April 1, 2018, through March 31, 2022. Of this cohort, 3% (n=458,049) were identified as immunocompromised (1). Overall, 13.5% (n=61,865) of this immunocompromised cohort developed COVID-19, with the largest prevalence among people with end-stage renal disease (20%), followed by primary immunodeficiency, hematopoietic stem cell transplant and solid organ transplant recipients (all 16%), immunosuppressive treatment (14%), and hematologic or solid tumor malignancy (9%). Of the immunocompromised cohort who developed COVID-19, 23.5% had hospitalizations associated with their first COVID-19 diagnosis, with a mean length of stay of 15.4 days. The mean cost for these events was estimated at nearly $1 billion US dollars in 2021, with a mean cost of $64,029 per patient.

INFORM, an ongoing retrospective cohort and electronic health data study in England (7), compared COVID-19–related outcomes (hospitalization, ICU admission, and death) among different groups of immunocompromised individuals and the general population. The study population comprised a random sample of 25% of all individuals aged ≥12 years (almost 12 million) registered in the National Health Service databases on January 1, 2022. Of this sample, 4% (470,910) were immunocompromised. Initial results from January 1 through December 31, 2022, showed that immunocompromised individuals disproportionately accounted for approximately 25% of COVID-related outcomes, including 22% (4585/20,910) of COVID-19 hospitalizations, 28% (125/440) of ICU admissions, and 24% (1145/4810) of deaths. Among the highly vaccinated population (≥3 doses of a COVID-19 vaccine), immunocompromised individuals accounted for approximately 25% of COVID-19 hospitalizations, 36% of ICU admissions, and 25% of deaths. In this group, the risks of COVID-19 hospitalization and ICU admission (incidence rate ratios adjusted for age, sex, and number of comorbidities) were 2.17 (95% CI 2.09–2.26) and 4.66 (95% CI 3.56–6.11), respectively. Individuals with the highest risk for COVID-19 hospitalization were solid organ and hematopoietic stem cell transplant recipients and those undergoing treatment for hematological malignancy.

### Indirect and non-clinical impact

**Figure 1:**
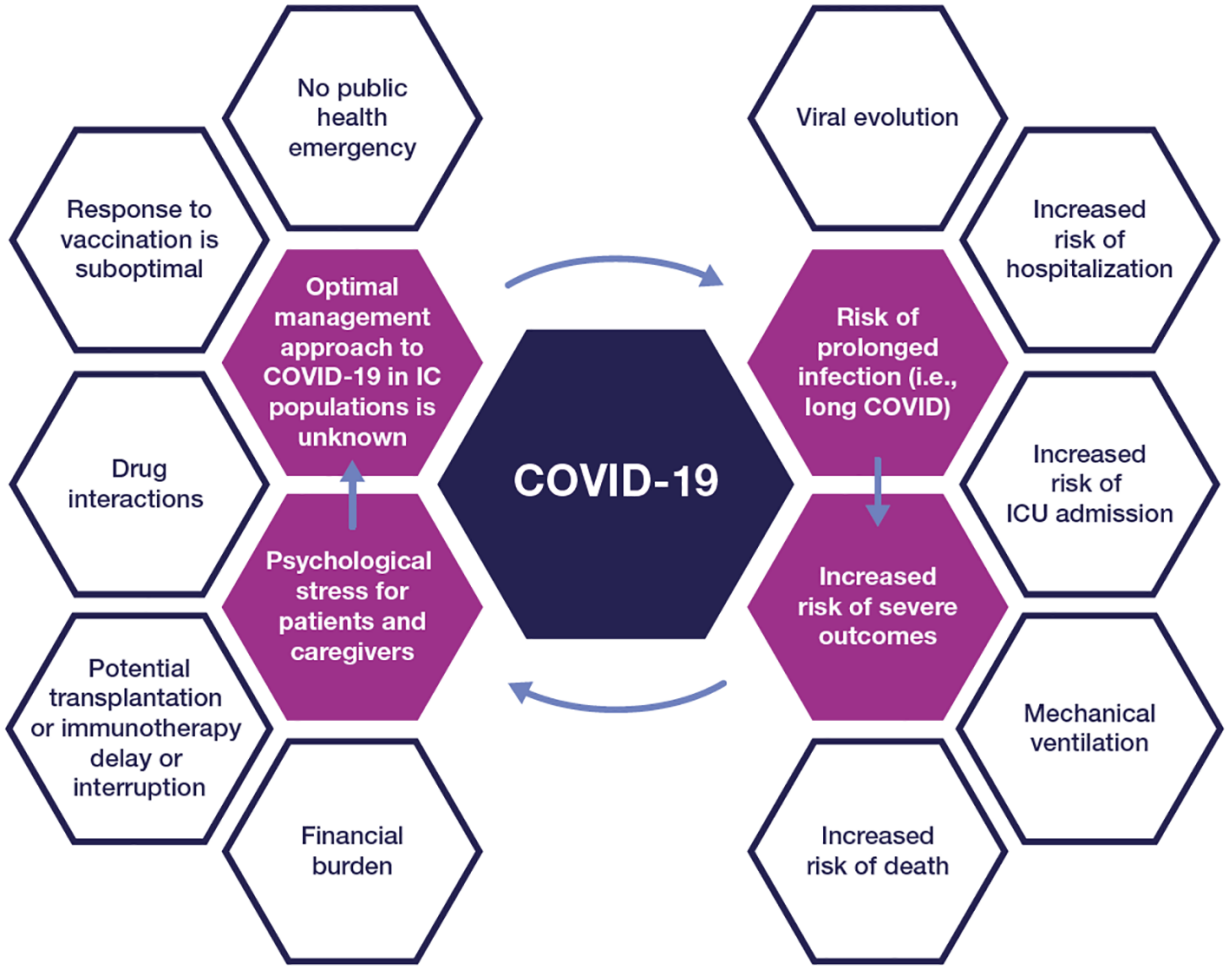
The impact of COVID-19 on people with compromised immunity IC, immunocompromised; ICU, intensive care unit.

Contracting COVID-19 can have additional negative consequences, including interrupted treatment for cancer or other underlying disease, potential adverse drug-drug interactions (DDIs) with COVID-19 treatments, loss of work productivity, social isolation, and emotional and financial burden (**Figure 1**). Furthermore, the initial infection could lead to a syndrome called “long COVID” with negative consequences in a person’s quality of life (14).

The emotional and psychological impact of COVID-19 on immunocompromised people, their families, and caregivers cannot be underestimated. A survey conducted at the beginning of the pandemic revealed high levels of fear/anxiety among 70% of adults with primary immunodeficiencies (15). These individuals were most afraid of exposure from contact with strangers, especially in public places. Qualitative analyses conducted among parents of children with cancer (16) revealed similar negative sentiments associated with COVID-19, while healthcare providers struggled with responsibility around inadvertently transmitting COVID-19 to their immunocompromised patients (17).

### Implications of viral persistence and evolution

Immunocompromised individuals with COVID-19 may suffer from persistent infection with prolonged viral shedding (18), and even viral rebound following treatment with antivirals (19), which carries significant public health implications. In a comprehensive review, DeWolf and colleagues (4) summarized the current understanding of how altered host immunity in individuals with cancer and other immunocompromising conditions impacts the prevention, clinical course, and long-term sequelae of SARS-CoV-2 infection. Multiple mechanisms contribute to the increased vulnerability of this population, depending on the type and severity of immunosuppression, including compromised epithelial barrier, impaired innate and adaptive T-cell immune responses, and altered tissue-resident immune cells (4). While antibody responses have been the primary clinical marker to prevent infection, T-cell responses also play a valuable role in controlling infection. Overall, the data suggest that neutralizing antibodies, CD4 T cells, and CD8 T cells work together in controlling COVID-19, and a deficiency in any one of the three immune measures is a risk factor for severe outcomes (20–23).

While immunocompetent individuals typically recover from COVID-19 within 5–7 days, immunocompromised people are at risk of prolonged infection due to slower clearance of the virus. People with hematologic malignancies and transplant recipients may shed viable virus for a median of 4 weeks (24–26). Moreover, SARS-CoV-2 may persist for an even longer period of time with proven infectivity (>8 months) (27), or progress into a chronic infection (long COVID-19) (28), increasing the opportunity for the emergence of mutant variants (29–31). A detailed analysis of an immunocompromised cohort with COVID-19 (18) showed that SARS-CoV-2 clearance and evolution varied by type and severity of immunosuppressive condition; suppression of both B- and T-cell responses resulted in the highest risk of persistent infection. Expectedly, individuals with hematological malignancies or hematopoietic stem cell transplantation were at increased risk of delayed viral clearance. Next-generation sequencing of available viral samples revealed that 39% of participants in the immunocompromised group had nucleotide changes in the spike protein vs. 12% of participants in the non-immunocompromised group, highlighting their potential as a source for mutant variants.

Indeed, the continuous rapid evolution of SARS-CoV-2 has had a substantial impact on the prevention and treatment of COVID-19. It has been challenging to predict how the virus may mutate, not only because of its unique biological signature as an RNA virus, but also because of its interactions with human and animal hosts (32, 33). Emergent variants have had different transmission patterns, virulence, and incubation periods (32, 34). Infections caused by Omicron variants are known to lead to less severe disease (35), but the short incubation period compared with previous variants (Omicron variant: 3.42 days [95% CI 2.88–3.96] vs. Alpha variant: 5.0 days [95% CI 4.94–5.06]) (36) could have contributed to faster and more widespread transmission. The JN.1 variant became dominant across the globe, with only one additional receptor-binding domain (RBD) mutation compared to its predecessor, BA.2.86 (37). Moreover, mutations at the N-terminal and RBD of the spike protein have led to a reduction or loss of the neutralizing activity of monoclonal antibodies (mAbs) against Omicron and its subvariants (38).

The BA.2.87.1 variant is now being closely tracked because it has over 30 changes in the spike protein of the virus when compared to XBB.1.5, the variant that the 2023–2024 vaccine was designed to protect against (39). The potential public health impact of these variants on the durability of vaccine and therapeutic coverage make it essential to address most, if not all, of the reservoirs of virus mutagenesis.

## Gaps in the current management landscape for immunocompromised people

### Vaccination response is suboptimal

Immunocompromised people respond to COVID-19 vaccination to different degrees but generally have a suboptimal response to vaccination and, consequently, less protection against severe outcomes (40). This is due to multiple factors, including reduced T-cell–specific or humoral-specific responses, which leads to low seroconversion rates post-vaccination, generation of antibodies with low neutralization activity against SARS-CoV-2 (41–43), and a short duration of protection. Studies have shown that although alterations in the B-cell compartment correlate with decreased humoral responses to COVID-19 vaccines, some degree of T-cell–mediated protection from severe disease might be conserved (23, 44, 45). A meta-analysis of 82 studies (46) showed that after one vaccine dose, people with hematological cancers, immune-mediated inflammatory disorders, and solid cancers were half less likely to seroconvert, while transplant recipients were 16 times less likely to seroconvert (risk ratio 0.06 [95% CI 0.04–0.09]) compared with immunocompetent controls. After a second dose, seroconversion remained least likely in transplant recipients. Parker et al. (47) identified 23 studies (2 clinical trials and 21 observational studies) reporting on vaccination outcomes for 1722 people with immunocompromised status. For participants with responses after the standard primary series, the median antibody response rate increased modestly from 41% (IQR 23–58) to 67% (55–69), while for low or non-responders, the median antibody response rate increased to 44% (32–55).

Waning of vaccine-induced protection is a major concern. Vaccine effectiveness decreases among individuals with comorbidities and those aged ≥55 years (48); this begins as early as the first month after full vaccination (defined as one or two vaccine doses, depending on vaccine type) (49). In immunocompromised individuals, the impact of waning is even more evident. During the Omicron period, vaccine effectiveness (defined as protection against COVID-19 requiring hospital admission following three doses of mRNA vaccines) in immunocompromised people waned to 48% (40–55%) by months four to five (50). By comparison, effectiveness in immunocompetent individuals decreased to 71% (68–74%). For the 2023–2024 (monovalent XBB.1.5) COVID-19 vaccine, effectiveness among adults aged ≥18 years with immunocompromising conditions was 38% in the 7–59 days after receipt of an updated vaccine dose and 34% in the 60–119 days after receipt of an updated dose (51).

### The public health emergency has ended

In May 2023, COVID-19 stopped being regarded as a global health emergency (52). The non-pharmacological interventions that had a positive impact on the course of the disease, such as free at-home rapid diagnostic tools, social distancing, and wearing masks, became less frequently implemented (53).

Additionally, uptake of the recommended vaccine boosters has decreased. As of March 31, 2024, only 23% (95% CI 22.1–23.1%) of adults in the United States reported having received an updated 2023–2024 COVID-19 vaccine (54). In particular, data from the VISION Network showed that only 18% of adults with immunocompromising conditions had received the updated COVID-19 vaccine (51).

### Prevention with mAbs has not always been available in the United States

A preventive strategy briefly available during the pandemic was the use of mAbs for pre- and post-exposure prophylaxis. This approach of passive transfer of immunity offered a more immediate and reliable level of neutralizing titers (55), especially for people who are unable to develop their own endogenous antibodies from vaccination or natural infection. In December 2021, the US Food and Drug Administration (FDA) granted emergency use authorization for tixagevimab-cilgavimab as pre-exposure prophylaxis in adults and children (≥12 years, weighing ≥40 kg) (56, 57). Real-world evidence studies (58–60) demonstrated the benefits of mAb prophylaxis in people with immunocompromised status. However, in January 2023, the emergency use authorization was retracted in the United States because of loss of neutralization activity against the Omicron XBB and subsequent variants (61, 62). Following that, until recently, there were no authorized mAbs for pre-exposure or post-exposure prophylaxis of COVID-19. On March 22, 2024, the US FDA authorized the emergency use of pemivibart (formerly VYD222) for pre-exposure prophylaxis among adolescents and adults with moderate-to-severe immune compromised status. The emergency use authorization was based on the totality of scientific evidence available, including demonstration of *in vitro* neutralizing activity against major SARS-CoV-2 variants, such as JN.1 (63), and immunobridging data from the ongoing CANOPY clinical trial (NCT06039449). Anaphylaxis was seen in four trial participants.

### Treatment with mAbs is not currently available in the United States

Therapeutic mAbs (e.g., bamlanivimab, bamlanivimab-etesevimab, casirivimab-imdevimab, sotrovimab, and bebtelovimab) are no longer authorized for the treatment of COVID-19 because of the loss of neutralizing activity against Omicron subvariants (64–66). This has limited the treatment options for some immunocompromised people who have impaired ability to mount an immune response to natural infection, especially those with CD20 depletion (67). Currently, there are limited data on potential future mAb therapies and their capacity to reduce the risk of disease progression in people with immunocompromised status.

Box 1 **Call to action: proposed strategies and considerations to improve COVID-19 outcomes for people with compromised immunity.**

**Table:** 

**Optimized vaccination strategies** – Establish the clinical efficacy of vaccines in terms of dosing and frequency – Address vaccine hesitancy – Increase vaccine uptake by target groups (e.g., vaccination campaign) – Continue development of vaccines against prevalent variants **mAbs to complement suboptimal vaccine responses** – Investigate potential mAbs for pre-exposure prophylaxis – Investigate potential mAbs for treatment – Support new technologies to predict the impact of emerging variants **Improved antiviral treatment** – Establish the dose, timing, and duration of treatment – Replace intravenous administration with oral administration – Limit the potential for DDIs with immunosuppressive treatments – Investigate effectiveness of combination treatment (e.g., with mAbs) **Public health emergency centered around immunocompromised people** – Implement public health measures (e.g., mask wearing, social distancing, isolation) – Support funding for new diagnostics, vaccines, and therapeutics	
	Additional considerations Logistics and infrastructure

### Current treatment regimens have limitations

Antiviral drugs available to treat high-risk individuals include remdesivir, nirmatrelvir/ritonavir, and molnupiravir (68). However, clinicians need to consider many factors when prescribing one of the three options. For example, nirmatrelvir/ritonavir and molnupiravir treatment must be initiated early (<5 days after diagnosis) (69, 70). Additionally, the use of ritonavir-boosted nirmatrelvir is associated with myriad of potential DDIs (71). Interestingly, there is reluctance among physicians in prescribing this antiviral combination drug for the immunocompromised population (72). Barriers to antiviral use among eligible patients with COVID-19 also include access, logistics, and lack of perceived effectiveness. Remdesivir is administered intravenously over a three-day treatment course (73), which could pose logistical challenges to the care team, while molnupiravir may not be as effective in reducing COVID-19 hospitalizations in patients at high risk of severe outcomes (74).

Another issue with antivirals for treatment of COVID-19 is the phenomenon of virologic rebound, characterized by recurrence of symptoms and reversion to SARS-CoV-2 test positivity after initial recovery with treatment. Estimates of viral rebound have varied; however, in a recent multicenter observational study of 127 participants (19), virologic rebound with shedding of replication-competent virus occurred in approximately 21% of people taking a 5-day course of nirmatrelvir-ritonavir and 2% of those who were not using therapy.

The use of convalescent plasma with high titers of anti–SARS-CoV-2 antibodies appeared to be a safe and effective way to treat severe COVID-19 at the onset of the pandemic. Although meta-analysis studies concluded that convalescent plasma was not associated with lower all-cause mortality or improved disease progression (75, 76), some National Institutes of Health panel members recommend the use of high-titer COVID-19 convalescent plasma, with or without antiviral therapy, for immunocompromised patients with prolonged, symptomatic disease (77). However, this treatment option is not widely available because of limited supply.

## Call to action: immunocompromised people need optimized prevention and treatment strategies

The limited options available to immunocompromised people for the prevention and treatment of COVID-19 represent a gap in the public health and therapeutic landscape. Large randomized clinical trials that led to the authorization and use of existing therapeutic agents often excluded immunocompromised participants, hence their benefits have not been rigorously tested in this group of vulnerable people (78). A definitive approach for the management of COVID-19 in people with immunocompromising conditions is missing. Given the lack of clear evidence-based studies, the current clinical approach has been extrapolated from larger trials focusing on non-immunocompromised individuals and case studies. A series of potential strategies that could improve outcomes for immunocompromised people are proposed (**Box 1**).

### Optimized vaccination strategies

Vaccination remains the cornerstone of prevention; however, since vaccination response is dependent on the individual’s immune system, an optimized strategy is needed for the immunosuppressed population. Depending on the COVID-19 vaccination history, different vaccination plans are subsequently recommended. People with immunocompromised status usually receive additional COVID-19 doses to boost their immune response at two or more months after the last recommended COVID-19 vaccine. The CDC developed specific guidance on COVID-19 vaccines for people who are moderately or severely immunocompromised (79), which are further endorsed by the National Institutes of Health (68). Improving the durability of vaccine response should also be prioritized. Considering that administration of the updated 2023–2024 COVID-19 vaccine resulted in only modest effectiveness (80), more research is needed in terms of dosing and frequency. Since there are different levels of immune compromise status and vaccine responses, it is suggested that dosing and frequency be individualized, as guided by underlying immune status and vaccine response.

### mAbs that complement suboptimal vaccine responses

There is an urgent need for the development of mAbs as prophylaxis for COVID-19 in people with immunocompromising conditions. This strategy is intended to provide passive immunity that complements the suboptimal vaccine response. Several mAbs are being studied in response to the emerging variants; two such long-acting mAbs (81, 82) are being evaluated for pre-exposure prophylaxis of COVID-19 in immunocompromised participants. Novel mAbs should be broad spectrum with a prolonged half-life and potential to treat emerging variants. New technologies that could predict emerging variants should be sought to guide the development of mAbs with a durable therapeutic lifespan.

### Improved antiviral treatment

Oral drugs are a preferred convenient approach to treatment, but they should be highly effective and without DDI potential. In addition, further consideration of the dose, timing, and duration of treatment with antiviral drugs is needed to inform optimal use and reduce the potential for viral rebound and infectivity. Various combination antiviral-antiviral drug or antiviral-antibody treatments have been explored. Small studies in B-cell–depleted patients with persistent SARS-CoV-2 infection showed better outcomes with combination therapy than monotherapy (83, 84). Mikulska et al. (85) investigated the combination therapy of remdesivir plus nirmatrelvir/ritonavir, or molnupiravir in case of renal failure, with mAbs in 22 immunocompromised patients with prolonged/relapsed COVID-19. The group observed significant increases in the rate of virological response when the combination treatment included mAbs, both at early stage (*P*=0.032) and after 30 days of treatment (*P*=0.046). However, controlled clinical trials are needed to advance these observations beyond case series and provide solid evidence for optimized antiviral treatment strategies.

### Targeted population-based health emergency centered around immunocompromised people

Healthcare systems, policymakers, and regulators need to work together and develop population-based health measures that focus on people with compromised immunity. Harm-reduction measures to prevent the spread of SARS-CoV-2 to the highest-risk individuals, such as masking and adherence to the CDC isolation guidelines, are known to be effective. A declaration of health emergency that is centered around immunosuppressed people will allow for more funding to develop new vaccines, diagnostics, and therapeutics for this vulnerable population.

## Conclusions

After more than four years since the start of the COVID-19 pandemic, SARS-CoV-2 has moved into an “endemic phase,” with periodic surges of cases throughout the year. Most people have either been infected or vaccinated, thereby having some level of protective immunity from severe disease. However, immune protection wanes over time, and this leads to a rise of cases and exposes vulnerable and immunosuppressed people to infection. This perspective provided a review of the gaps in the management of COVID-19 among immunosuppressed individuals. A call to optimize prevention efforts through vaccination and passive immunity transfer is highlighted. Likewise, improvement in treatment strategies, such as development of novel, safer drugs, and the exploration of combination therapies, is needed. Such efforts to optimize the care of immunosuppressed individuals should be supported by public agencies in partnership with the healthcare industry and institutions.

## Data availability statement

The original contributions presented in the study are included in the article. Further inquiries can be directed to the corresponding author.

## Author contributions

RR: Conceptualization, Investigation, Writing – original draft, Writing – review & editing.
