# Corrigendum: Protecting the vulnerable: addressing the COVID-19 care needs of people with compromised immunity

**DOI:** 10.3389/fimmu.2024.1440571

**Published:** 2024-07-25

**Authors:** Raymund R. Razonable

**Affiliations:** ^1^ Division of Public Health, Infectious Diseases and Occupational Medicine, Department of Medicine, Mayo Clinic, Rochester, MN, United States; ^2^ William J. von Liebig Center for Transplantation and Clinical Regeneration, Mayo Clinic, Rochester, MN, United States

**Keywords:** COVID-19, immunocompromised, SARS-CoV-2, prevention, treatment

In the published article, there was an error in the **Funding** statement. The published Funding statement stated that “The author declares financial support was received for research, authorship, and/or publication of this article”, which is not correct. The correct **Funding** statement appears below.

**FUNDING**

The author declares no financial support was received for research, authorship, and or publication of this article. The article processing fee was paid by Invivyd, Inc.

The authors apologize for this error and state that this does not change the scientific conclusions of the article in any way. The original article has been updated.

